# Asymmetrically Nanostructured 2D Janus Films Obtained from Pickering Emulsions Polymerized in a Langmuir–Blodgett Trough

**DOI:** 10.3390/mi14071459

**Published:** 2023-07-20

**Authors:** Andrei Honciuc, Oana-Iuliana Negru

**Affiliations:** “Petru Poni” Institute of Macromolecular Chemistry, Aleea Gr. Ghica Voda 41A, 700487 Iasi, Romania

**Keywords:** Langmuir–Blodgett, self-assembled nanoparticles, 2D films, Pickering emulsions, Janus films, surface nanostructuring

## Abstract

Low-dimensional structures, such as two-dimensional (2D) Janus films, can be useful in studying fundamental interactions or in applications at the nanoscale. In this work, we report the fabrication of 2D polymer Janus films consisting of one smooth and another nanostructured facet on which silica nanoparticles (NPs) are self-assembled in a compact monolayer shield. The 2D films are made from Pickering emulsions of monomers in water, stabilized by NPs, which are spread over the surface of the water in a Langmuir–Blodgett trough. Following the spreading of the colloidosomes, oil droplets stabilized by NPs collapse, and the interfaces reorganize such that the NP monolayer is found exclusively at the oil/water interface. Upon compression followed by UV polymerization, a 2D solid film is formed, with one smooth and another nanostructured face. The film can be removed from the surface of the water and handled with tweezers. The 2D films exhibit different surface properties on the two sides, such as differences in water wettability. On the nanostructured side, water wettability can be tuned by tuning the surface energy of the nanoparticles, namely by changing their surface functional groups. Upon removal of NPs, the surface can be patterned with an array of circular traces.

## 1. Introduction

The construction of 2D films or membranes can be performed either using “top-down” or “bottom-up” methods [1]. The bottom-up fabrication methods rely on the natural tendency of several building blocks to come together through the process of self-assembly [2,3]. For example, surfactants and amphiphiles self-assemble at the air–liquid or liquid–liquid interfaces, forming supported 2D films whose thicknesses are negligible compared to their lateral dimensions. This happens most commonly with surfactant molecules that self-assemble at the interface between two phases in emulsions, in foams, or in self-assembled monolayers. In a similar way, nanoparticles that are pseudo amphiphilic or amphiphilic, thus resembling molecular surfactants [4,5], can self-assemble at liquid–liquid interfaces in Pickering emulsions or air–liquid interfaces in Pickering foams, forming a 2D film or nonporous membrane, respectively. Populating an interface with surfactants or nanoparticles can fundamentally change its physicochemical properties by changing its cohesion, surface tension, permeability, etc. Sometimes, by removing one participating phase, the self-assembled structure formed at the interface can be revealed if it is sturdy enough, thus transforming it into a supported film or membrane and a free-standing membrane if both phases are removed [6]. For example, 2D free-standing mono and bilayers constituted of self-assembled Janus nanoparticles can be obtained by draining the water from the Pickering foam, revealing the intimate process of interfacial self-assembly of these amphiphilic nanoparticles [6] that resemble molecular surfactants [7]. Another example is the polymerization of Pickering emulsions, where the oil phase is a monomer that can be polymerized, resulting in surface nanostructured microspheres [8] or microporous foams [9]. In these cases, the surface nanostructuring of the resulting polymer surface is a consequence of trapping at the interface of the nanoparticles that stabilize the Pickering emulsions [10,11,12]. Further, transformations are possible, leading to various levels of interfacial nanostructuring that can take place if chemical transformations take place at the liquid–liquid interfaces, that is, if reactants or products can be transferred unidirectionally across the interface. For example, in a process resembling a chemical garden, due to the compartmentalization of reactants in either water or the organic phase of Pickering emulsions and interfacial polymerization with unidirectional transport of monomers across a self-assembled monolayer of nanoparticles, an asymmetric nanostructured film was obtained, with a honeycomb interfacial nanostructure on one side and nano protrusions on the other side [13]. In a larger context, any asymmetric transformations taking place at the interfaces or on each side of a film can lead to the formation of an asymmetric film in both chemistry and surface morphology. Such asymmetric films are called “Janus” films or membranes, after the Roman deity with two faces. These proved useful in a plethora of new applications, such as elements for self-adaptive and pneumatic multifunctional electronics [14], triboelectric nanogenerators [15], fabrication of membranes with asymmetric wettability, but also in typical membrane applications microfiltration, ultrafiltration [16], membrane distillation, reverse osmosis, etc., showing superior performance in oil/water separations because, in theory, a Janus membrane can spontaneously achieve unidirectional liquid transport [17,18,19]. 

In this work, we show a new method of preparing a 2D asymmetrically nanostructured Janus film from a Pickering emulsion that was first spread on the surface of water and then polymerized. Pickering emulsions are emulsions stabilized by nanoparticles, and they can be oil-in-water (o/w), with the dispersed phase being oil, or water-in-oil (w/o), with the dispersed phase being the water. In this case, the oil is a vinyl-bearing monomer that is immiscible with water. The thus formed Pickering emulsion was spread on the surface of a Langmuir–Blodget trough. Upon contacting the water surface, a rearrangement of the nanoparticle shield of the emulsion droplet occurred such that the monomer droplets collapsed, forming a contiguous film, and the stabilizing nanoparticles ended up only on the monomer/water interface. Upon polymerization, the surface nanostructured Janus polymer film can be transferred onto various substrates or handled with tweezers. To the best of our knowledge, this facile and straightforward method for preparing Janus polymer films that are nanostructured on one side and smooth on the other side has not been reported. Further investigations reveal that the obtained Janus films exhibit stark differences in water wettability on the two facets due to the surface nanostructuring and surface polarity of the nanoparticles. Initially, the nanostructured part of the Janus film shows dynamic wetting behavior due to a transition from one wetting regime to another with an overall hydrophilic wetting behavior. The smooth side is rather hydrophobic and exhibits high water contact angles. Additional surface treatment of the nanostructured surface with a hydrophobizing agent transforms the nanostructured side into a superhydrophobic surface. Due to their asymmetry and contrasting properties, these Janus films can be used in a variety of applications, ranging from stabilizing foams and emulsions to coatings for fouling mitigations or nano-filtration applications. Further, the hydrophilic peaks of the nanostructured side resemble the surface structuring of the Namib-desert beetle, whereas its hydrophilic peaks and hydrophobic valleys [20,21] make it an ideal candidate for potential use as a biomimetic coating for fog harvesting. 

## 2. Materials and Methods

### 2.1. Materials

Tetraethylorthosilicate (TEOS) 99%, benzoin methyl ether (BME) 97%, trimethoxy(octyl)silane 97% (OTS), and 3-(mercaptopropyl)trimethoxy silane (MPTMS) were purchased from ABCR GmbH (Karlsruhe, Germany). 3-(triethoxysilyl) propionitrile 97% (TESPN), 3, (3-glycidoxypropyl)trimethoxysilane (GLYMO), divinylbenzene (DVB) technical grade 80%, tert-butyl acrylate (tBA) 98% containing 10–20 ppm monomethyl ether hydroquinone as inhibitor, aluminum oxide (active basic) Brockmann I, and hexamethyldisilazane (HMDS) were purchased from Sigma-Aldrich (Merck KGaA, Darmstadt, Germany). Absolute ethanol (EtOH, 99.3%) and hydrochloric acid (HCl) were purchased from Chemical Company (Iași, Romania). Ammonium hydroxide solution NH_4_OH (28–30%) EMSURE ACS. Reag. Ph Eur was purchased from Sigma-Aldrich (Merck KGaA Darmstadt, Germany). Supelco, Hostasol Yellow 3G dye (Clariant, Wiesbaden, Germany) was generously donated by HSH Chemie S.R.L.

### 2.2. Synthesis of Silica Nanoparticles

The preparation procedure for silica nanoparticles (NP) and silica nanoparticles functionalized with nitrile, NP-CN by reaction with TESPN, alkyl, NP-C8 by reaction with OTS, thiol, and NP-SH by reaction with MPTMS were reported previously [10], and the functionalization with epoxy (NP-Gly) by reaction with GLYMO was also reported [11]. Briefly, 6 mL TEOS, 300 mL EtOH, 22 mL H_2_O, and 18.5 mL NH_4_OH were added into a 1000 mL round bottom flask, and the mixture was stirred at room temperature at 1000 rpm. Next, a mixture of 36 mL TEOS and 150 mL EtOH was added dropwise via a separatory funnel and left under stirring for 20 h at room temperature. After this period, the reaction mixture was neutralized with 12 mL of HCl. The silica nanoparticles were washed in this way three times with EtOH and three times with water, and the separation from the solvent was performed by centrifugation for 4 min at 5500 rpm with a Hermle Z 326K centrifuge at 0 °C. In the end, the particles were dispersed and stored in EtOH. The average diameter of the obtained NPs was D = 500 ± 7 nm, determined with SEM.

For surface functionalization, 1.2 g of obtained silica nanoparticles dispersed in 20 mL of EtOH was poured in a 100 mL flask. Then, 30 mL of EtOH was added, and the mixture was kept under an Ar atmosphere and stirred at 1000 rpm. Next, 2 mL of silane coupling agents OTS, MPTMS, TESPN, or GLYMO was added dropwise. Next, the reaction mixture was heated and maintained to 60 °C for 20 h. The functionalized nanoparticles were washed three times with EtOH, three times with H_2_O, two times with EtOH, and another two times with H_2_O, and were finally redispersed in H_2_O.

### 2.3. Pickering Emulsion Preparation 

Water-immiscible tBA containing 0.1% Hostasol Yellow 3G and a crosslinking monomer DVB were used for the Pickering emulsion preparation and polymerization. Pickering emulsion was produced by first adding 20 mg of BME radical initiator to a 20 mL glass scintillator vial, followed by 1 mL of monomer, 0.1 mL of DVB crosslinker, and 5 min of waiting for the mixture to become homogeneous. Next, 5 mg of colloidal particles and 12 mL of water are added. The glass scintillator vial was then sonicated with a Branson 450 Sonifier equipped with a 7 mm diameter horn for 15 s at 30% amplitude. 

### 2.4. Film Preparation at the Surface of the Langmuir–Blodgett Trough and Polymerization

The Langmuir–Blodgett (LB) experiments were carried out with a Kibron Microtrough G1, equipped with a liquid/liquid o/w trough from Kibron Inc. Finland. The oil in water (o/w) emulsions, where the oil is represented by the tBA monomer (containing 0.1% Hostasol Yellow 3G), are gently spread on the surface of water contained in the Kibron LB trough, with the help of a spoon spatula, approximately 0.5 mL Pickering emulsion. During spreading, the barriers of the LB trough are fully open, 100% area, and after film spreading, the barriers are closed, compressing the film until the area is approximately 10–20%. At this point, the barriers are stopped, and the monomer film is exposed for 2 h to a UV lamp (wavelength = 365 nm, with 4 lamps, each with an intensity = 2.2 mW/cm^2^). After the polymerization, the polymer film is collected from the water surface either on a wire frame for further handling and processing or on an aluminum stub for scanning electron microscopy (SEM) investigations. 

The nanoparticles trapped at the surface of the film can be removed by treatment with NaOH 10 M for 24 h, after which the films are removed and washed.

### 2.5. Two-Dimensional Film Characterization

#### 2.5.1. Contact Angle Measurement

The contact angle of a sessile water droplet was measured on a 2D film immobilized on carbon tape with an OCA 15EC/B video-based contact angle measuring instrument (DataPhysics GmbH, Filderstadt, Germany) equipped with a video camera and SCA 20 software capable of automatic detection of the contour of the droplet and measurement of the contact angle.

#### 2.5.2. Scanning Electron Microscopy

The 2D films were investigated with a Verios G4 UC (Thermo Fischer Scientific Inc., Eindhoven, The Netherlands) scanning electron microscope (SEM) with beam energy of 5 keV using an Everhart-Thornley detector, beam spot 50 pA. 

## 3. Results 

The starting silica NPs were synthesized according to a previously reported method [10], a modified version of the Stöber process. Also, the preparation of functionalized nanoparticles bearing nitriles, NP-CN, alkyl NP-C8, and thiol NP-SH, and glycidyls, NP-Gly, were reported elsewhere [10,11,22]. Pickering emulsions were prepared according to the reported recipes by ultrasonication of tBA monomer stabilized by various NPs. In a Pickering emulsion, the oil droplets of the dispersed phase stabilized by nanoparticles are usually referred to as “colloidosomes”.

### 3.1. Preparation of the 2D Film from Pickering Emulsions on the Langmuir–Blodgett Trough

**Figure 1:**
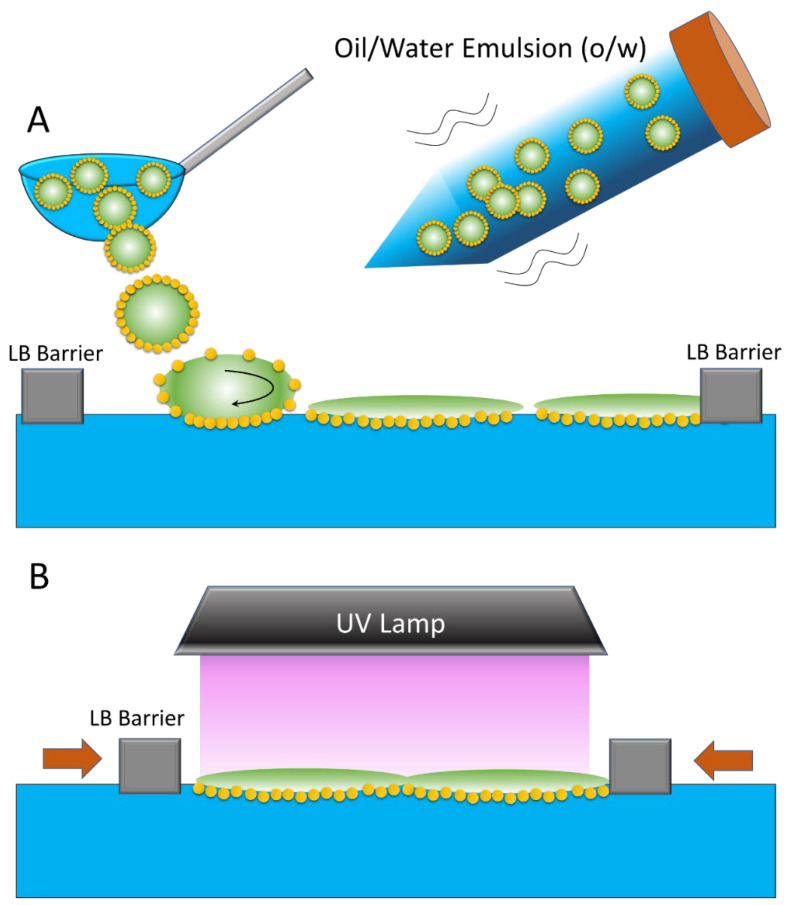
(**A**) Cartoon depicting the preparation of the 2D film on the surface of water on a Langmuir–Blodgett trough by spreading the o/w emulsion, where the oil is the tBA monomer, followed by (**B**) compression of the barriers and the exposure to UV light. The lateral arrows indicate the film compression by the barrier.

2D films were prepared by spreading the Pickering emulsions of the tBA monomer stabilized by various nanoparticles, NP-CN, NP-C8, NP-SH, and NP-Gly, according to Figure 1. In the first step, the o/w emulsion was prepared from the monomer tBA in water and stabilized by NPs. Next, the emulsion was gently spread on the surface of the water with the help of a spoon spatula, as in Figure 1A. The tBA monomer has a lower density (*ρ* = 0.85 Kg/L) than water, and consequently, the tBA colloidosomes float on the water surface, exhibiting a high surface mobility (see Movie S1), which continues for several minutes until settling and collapsing into a film on the surface of the water, with the NPs distributed at the bottom, i.e., at the contact with water, as in Figure 1A. We hypothesize that the high surface mobility of the droplets, as observed in the film, could be due to the time taken by NPs to re-distribute at the monomer/water interface under the action of capillary forces, surface tension gradients, and other hydrodynamic forces. The highly dynamic colloidosomes, rapidly moving on the surface of the water, can be used as fuels for the propulsion of various objects on the surface of the water. Following this re-distribution, we observe that the NPs are found exclusively at the monomer/water interface, as depicted in Figure 1A, and there were none at the monomer/air interface, as we will be showing next in the electron microscopy studies. After the monomer is evenly spread on the surface of the water, the Langmuir–Blodgett barriers are compressed until the surface area of the film decreases from 100% to 10–20%. During compression, the individual droplets of monomer are brought together, and these slowly coalesce into a contiguous liquid monomer film floating on top of the water surface. During the compression, the surface tension is continuously monitored, and this decreases to lower values than that of water and asymptotically towards the tBA/water interfacial tension of 57 mN/m, signaling that a fully formed interface between the two fluids formed. When the value of the interfacial tension of approximately 57 mN/m was reached, the barriers were stopped, and the film was next exposed to UV radiation from a lamp for cca. 2 h, during which the polymerization began, and the monomer film was slowly converted into a polymer film. 

After preparation, the polymer film can collected from the surface water in various ways: by a wire frame formed from a paper clip, directly on the SEM aluminum stub, or on flat microscope glass substrates or coverslip for contact angle measurements. 

**Figure 2:**
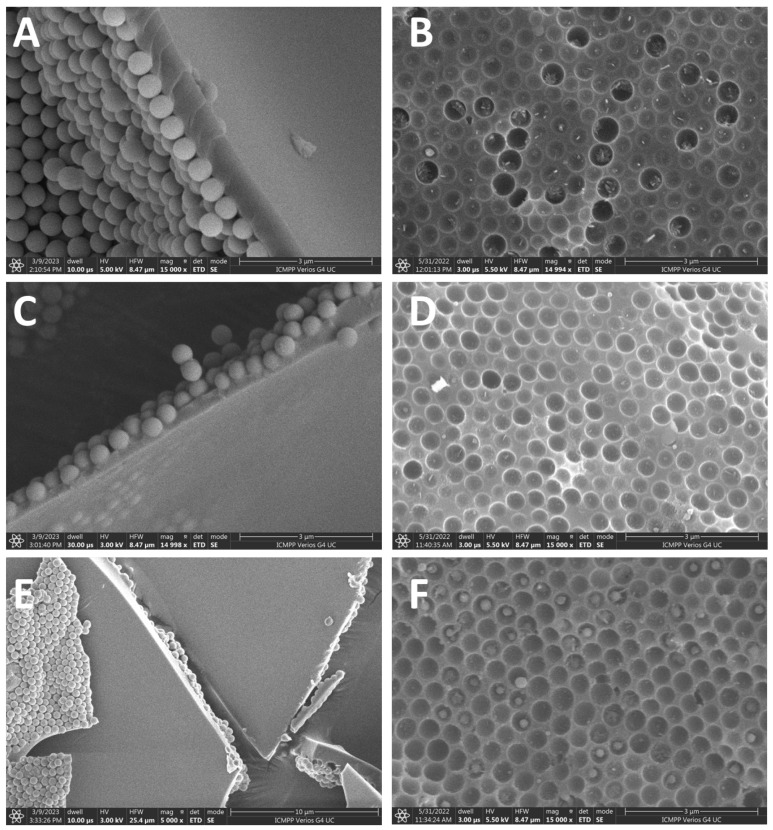
SEM images of the 2D-films of PtBA stabilized by different nanoparticles before treatment with the NaOH (**left**) and after (**right**): (**A**,**B**) NP-SH, (**C**,**D**) NP-C8, and (**E**,**F**) NP-CN.

The obtained 2D films resulted from the polymerization of the water-spread o/w Pickering emulsions, whereas the tBA was the dispersed phase stabilized by NP-SH, NP-C8, and NP-CN on the surface of the Langmuir–Blodgett trough, as shown in Figure 2. From the SEM images, we can see that the film thickness is comparable to the diameter of the NPs, which is around 500 nm, as seen in Figure 2A,C,E. Upon removal of the NPs, the surface nanostructuring changes to an array of circular traces, as seen in Figure 2B,D,F. In Figure 2B,D,F, some circular traces appear bigger than the others; we believe this is the result of the jamming phenomenon we observed and discussed previously [10], where some of the particles are pushed out of the packing plane, and thus create a bigger hole in the surface. This is further amplified by the treatment of the membrane with hydroxide, and in fact, this should be the path to the opening of the pores in the film to create a porous membrane.

Further, it can also be seen that the 2D film is asymmetrically structured, meaning that only one side has nanoparticles, self-assembled into a compact monolayer, while the other is completely empty of nanoparticles. We hypothesize that the rearrangement of NPs occurs because of the action of the capillary forces acting onto the NPs during the water draining while spreading the o/w Pickering emulsion onto the surface of the water; see Figure 1B. 

### 3.2. Adjusting the Film Thickness 

**Figure 3:**
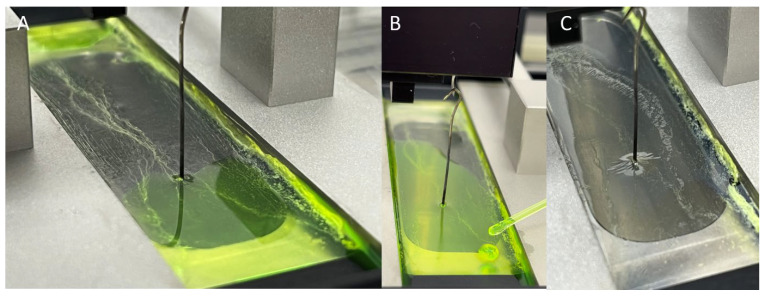
The adjustment of the Janus film thickness by loading the Janus 2D film, formed by spreading the Pickering emulsion on the surface of the water and compression (**A**), additional liquid monomer (**B**), and polymerization into a thin film membrane (**C**). For better visualization of oil, only Hostasol Yellow 3G dye was dissolved into the tBA monomer.

The film formed by spreading the Pickering emulsion stabilized by NP-Gly on the surface of the LB trough, upon compression of the barriers, can sustain an additional liquid layer, such that on a surface area of 2 cm × 8 cm, 2 mL of liquid tBA could be added. In Figure 3, this process is shown, whereas the additional liquid monomer is added with a pipette drop by drop up to 2 mL, and then the film is kept under the pressure of the lateral barriers of the LB trough and polymerized by exposure for 2 h to UV-light. In this way, the thickness of the Janus film can be adjusted from about 500 nm, which is the initial thickness obtained by only spreading the Pickering emulsion to up to several microns by adding a supplementary amount of tBA monomer after the initial film is formed. 

**Figure 4:**
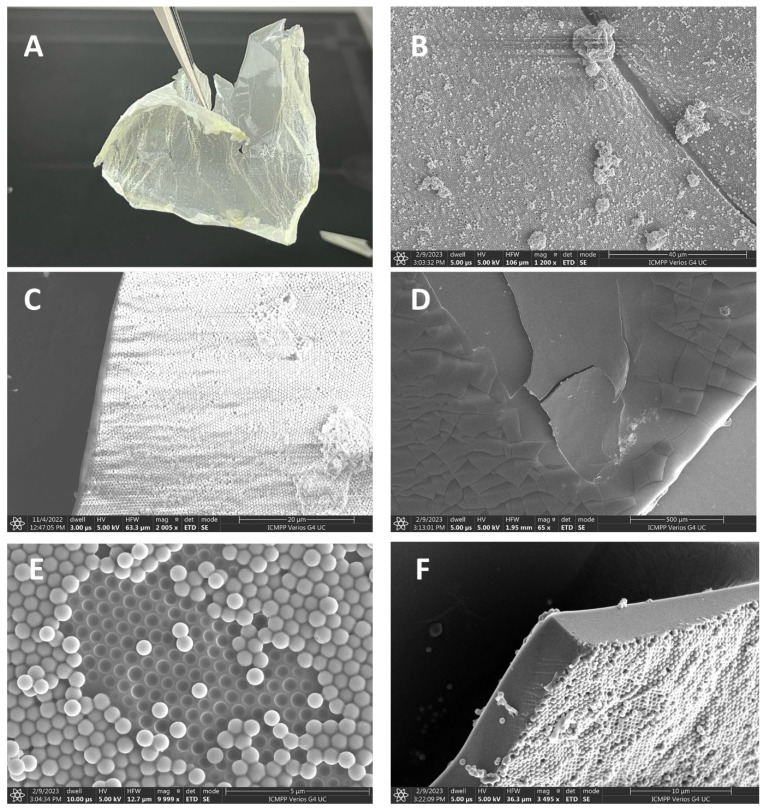
(**A**) Photograph of the PtBA Janus films obtained on the surface of the LB trough from a Pickering emulsion stabilized by NP-Gly; (**B**) SEM image of the bottom of the film (film that was in contact with water) showing a rough surface due to the presence of NP-Gly, nanoparticle clusters, and polymer protrusions; (**C**) SEM images of the film showing large scale NP-Gly assembly and hexagonal compact packing; (**D**) SEM image of the top of the film (the film that was in contact with the air), showing a smooth surface and the absence of NPs; (**E**) hexagonal close packing (HCP) of the NP-Gly on the bottom side of the film; and (**F**) the cross-section of the PtBA film.

The thick polymer film can be collected from the surface of the water in different ways: with tweezers, such as in Figure 4A, scooped with a glass coverslip, or collected with a wireframe from a paperclip. Analyzing this film via electron microscope keeps its asymmetry in the way that the nanostructuring on one side, Figure 4B,C,E,F, on the water side is preserved, while on the top side, Figure 4D,F, the air side, this polymer film is perfectly smooth. Because of the non-negligible lateral dimensions of this film, this is called a Janus film. We also note that the dye used for visibility has no influence on the results obtained, as exactly the same results are obtained in its absence.

### 3.3. Measuring the Contact Angles of the Functionalized NPs on the Polymer Films

**Figure 5:**
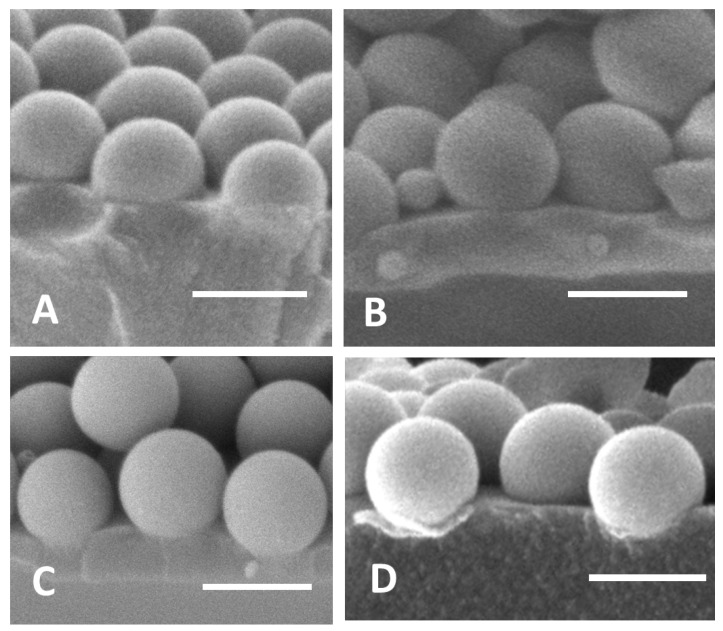
(**A**) NP-CN, *β* = 88°, (**B**) NP-C8, *β* = 124°, (**C**) NP-SH, *β* = 129°, and (**D**) NP-Gly, *β* = 94°. The scale bar in the images is 500 nm.

The contact angle of individually functionalized nanoparticles can be measured directly from the cross-section of the film, as shown in Figure 5 for each nanoparticle. To the best of our knowledge, this is the only direct way to measure the contact angle of the NPs with a polymer. The values of the contact angles increase in the order of NP-CN: *β* = 88°, NP-Gly: *β* = 94°, NP-C8: *β* = 124°, NP-C8: *β* = 124°, and NP-SH: *β* = 129°, which are quite different from values measured in a previous work based on the NanoTraPPED method. For a comparison between these values, see Table 1. The difference between the values of the contact angles observed here and the NanoTraPPED method indicates that the process of Pickering emulsion droplets, upon spreading on the surface of the water, go through a process of deconstruction and rearrangement of the water/monomer interface. 

## 4. Discussion

The surface properties of the 2D nanostructured films differ on the two sides. This is to be expected due to several factors: (i) nanostructuring and (ii) the effect of the nanoparticle surface properties. This can be evidenced by the different water-wetting behaviors that each face of the film exhibits. Creating the so-called Janus films with a difference in wettability on two faces is especially important for employing these films in applications such as emulsification, foaming, filtration, desalination, or biomedical applications [18].

**Figure 6:**
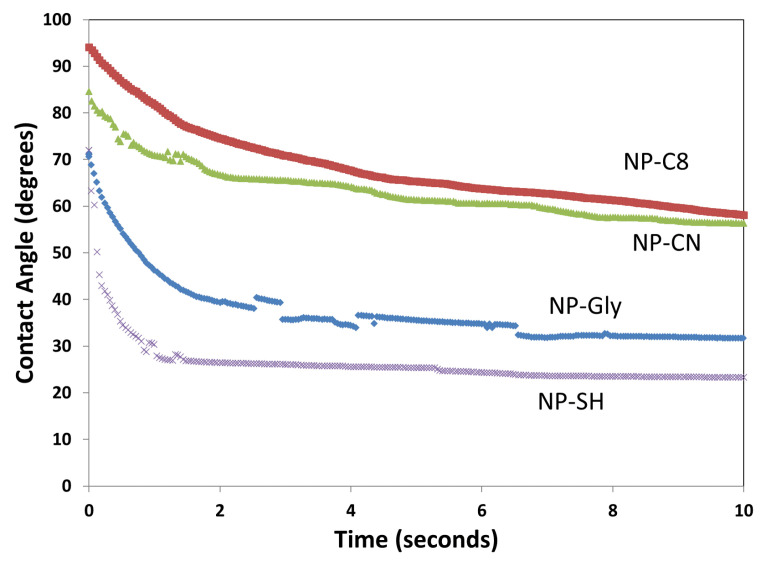
Evolution of the contact angle of a water sessile drop on the nanostructured side of the 2D film.

The dynamic water contact angle was measured via the sessile drop method both on the nanostructured and the smooth side of the asymmetrically structured 2D films. The water contact angle measurements performed on the smooth side, the side without nanoparticles, exhibit an equilibrium value of 74–78°; see Figure S1 in Supplementary Information (SI), which is the typical value obtained for the PtBA polymer. The water contact angle measurements performed on the nanostructured side of the film are presented in Figure 6, and it shows that the liquid droplet on the nanostructured side of the 2D films is at non-equilibrium following the deposition and quickly spreads to an equilibrium value given by the plateau. 

**Figure 7:**
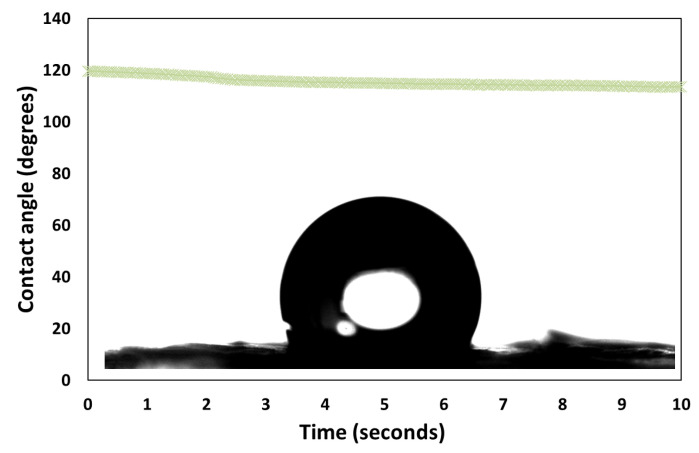
Evolution of the contact angle of a water sessile drop on the HMDS-treated nanostructured side of the 2D film. The inset shows a snapshot of the sessile droplet on the HMDS-treated nanostructured surface.

Given the nanostructured nature of the 2D film, as in Figure 4 and Figure 5, the water spreading behavior can be explained first by a transition between the Cassie–Baxter to Wenzel wettability state, as explained in reference [1]. The plateau value decreases for the 2D films on the nanostructured side in the following order: NP-C8 (≈65°), NP-CN (≈61°), NP-Gly (≈35°), and NP-SH (≈25°). This correlates well for the first three with the surface energy of the nanoparticle and especially with the polar surface energy component, that is, the surface polarity, which increases in the same order for the above nanoparticles, NP-C8 (≈14 mJ·m^−2^), NP-CN (≈27 mJ·m^−2^), and NP-Gly (≈42.5 mJ·m^−2^) [10,11]. This suggests that the surface energy of the nanoparticles, i.e., surface polarity, is the main parameter controlling the speed of transition from a Cassie–Baxter state to a Wenzel state, but also the equilibrium value of the water contact angle; see Figure 6. The fact that the equilibrium value of the water contact angle on the nanostructured side changes to lower values with the increase in polarity of the nanoparticle surface can be understood by considering the transition of the sessile droplet from a pure Cassie–Baxter state to a superposition of Cassie–Baxter and Wenzel states, as previously observed for Janus films or membranes [23]. In other words, because the chemical nature and the surface properties of the nanoparticles differ from that of the polymer substrate, both the chemical heterogeneity and surface roughness must be considered in the final state at equilibrium; thus, in the current situation, the final state can be seen as a combination of the Cassie–Baxter and Wenzel states. In fact, it was recently shown [24] that, indeed, a transition from the Cassie–Baxter state to a Wenzel state transition produces a continuous spectrum of a linear combination between the two basis states. To prove that the main parameter controlling the wettability observed on the nanostructured side is the surface polarity of the nanoparticles, we treat the surface with hexamethyldisilazane (HMDS), a well-known surface hydrophobizing agent. The water contact angle of the HMDS-treated nanostructured surfaces is presented in Figure 7. In this case, the values of the water contact angles increased significantly and are at an equilibrium value of between 116 and 130° for all the substrates, as in Figure 7 and Figure S2. This suggests that the hydrophobization of the NPs and the polymer substrate inhibits the transition from the Cassie–Baxter state to the Wenzel state, whereas the droplet finds itself much closer to the pure situation of a Cassie–Baxter state, leading to superhydrophobic films. The hydrophobization of both NPs and the polymer “switches off” the transition between the two states, locking the water droplet into a Cassie–Baxter state. This was the case for all the substrates, which exhibited the same contact angle after hydrophobization, except for the NP-C8 nanostructured film, for which a lower contact angle was obtained; see Figure S2. The hydrophobization with HMDS NP-C8 nanostructured film was probably not successful due to the reduced reactivity of alkyl groups already present on the surface of the NPs. 

Apart from the interesting fact that we provided a way to tune the surface wettability on the nanostructured side, we have also provided a new practical way to produce a superhydrophobic film. In fact, with the help of the Langmuir–Blodgett trough and dip coating technique, these Janus films could be easily deposited on any surface, such as glass slides, fibers, and objects of various shapes, to achieve biomimetic coatings on various materials for fog harvesting applications [21], icephobic coatings [25], omniphobic coatings [26,27], etc. From our tests, the Janus 2D films have a good adhesion onto glass and metallic substrates and do not easily peel off or flake off upon immersion into a low-surface tension liquid. 

## 5. Conclusions

In this work, we demonstrated a facile way to obtain asymmetrically nanostructured 2D Janus films by first spreading a Pickering emulsion of a monomer stabilized by NPs and then polymerizing it with UV light. The obtained 500 nm thick Janus film shows significant differences in wettability between both faces. On the nanostructured side, a sessile water droplet spreads until it reaches a plateau, suggesting that it undergoes a transition from the Cassie–Baxter state to the Wenzel state in less than 10 s. This film wettability on the nanostructured side can be tuned by tuning the surface energy of the NPs by changing the surface functional groups on the nanoparticles. The more polar the NPs’ surface functional groups, the more wettable these become. However, when the Janus film is further hydrophobized with HMDS, superhydrophobicity is obtained. On the other side, the contact angle is constant, and it corresponds to the water contact values typically measured for this polymer, and this cannot be tuned. In this way, the contrast in wettability between the two sides of the film can be tuned, which is important for a plethora of applications, ranging from the manufacturing of membranes for water distillation to desalination, emulsification, and other biomedical applications. The facile synthesis, versatility of coating the 2D Janus film on various substrates, and ability to tune the wettability contrast between the two sides from a superhydrophobic to hydrophilic state make the 2D Janus film a viable candidate for a plethora of existing and new applications.

## Figures and Tables

**Table 1:** Comparison between the contact angle values of the nanoparticle with the polymer by two different methods, NanoTraPPED and the current method, by the analysis of the cross-section of the film, as seen in Figure 5.

Nanoparticle	Current Method (°)	* NanoTraPPED (°)
NP-CN	88	119.4 ± 1.8
NP-Gly	94	153.47 ± 0.92
NP-C8	124	115.4 ± 3.2
NP-SH	129	115.7 ± 2.6

* Values taken from references [10,11].

## Data Availability

The data generated in this study are publicly available in an open access repository, the Open Science Framework (OSF) repository at DOI 10.17605/OSF.IO/TYM28.

## Supplementary Materials

The following are available online at https://www.mdpi.com/article/10.3390/mi14071459/s1. Figure S1: Contact angle on the smooth side of the Janus film. Figure S2: Contact angles of water on the HMDS-treated Janus films. Table S1: A summary of the conditions used in the colloidosome synthesis. Movie S1: Spreading of the Pickering emulsion on the surface of the water in the Langmuir-Blodgett trough.
