# Supplementary material for: Asymmetrically Nanostructured 2D Janus Films Obtained from Pickering Emulsions Polymerized in a Langmuir–Blodgett Trough
Source: Micromachines (Basel). 2023 Jul 20;14(7):1459. doi: 10.3390/mi14071459 (PMC10384703; doi:10.3390/mi14071459)
Supplement: Supplementary file 1 [file micromachines-14-01459-s001.zip › 2023.05.24_SI Nanostructured 2D Membranes AH.pdf]

Supplementary Information

Asymmetrically Nanostructured 2D Janus Films Obtained from Pickering Emulsions Polymerized in a Langmuir-Blodgett Trough

Andrei Honciuc*, Oana-Iuliana Negru

“Petru Poni” Institute of Macromolecular Chemistry, Aleea Gr. Ghica Voda 41A, Iasi, 700487, Romania

* Correspondence: honciuc.andrei@icmpp.ro

Table S1. A summary of the conditions used in the colloidosome synthesis.

**Monomer**	**Functional Group**	**DVB [mL]**	**BME [mL]**	**H_2O [mL]**	**NPs [mg/mL]**	**Sonication Time (s)**	**Amplitude**
tBA - 1	NP-CN	0.1	20	12	5	15	30
	NP-C8	0.1	20	12	5	15	30
	NP-SH	0.1	20	12	5	15	30
	NP-Gly	0.1	20	12	5	15	30

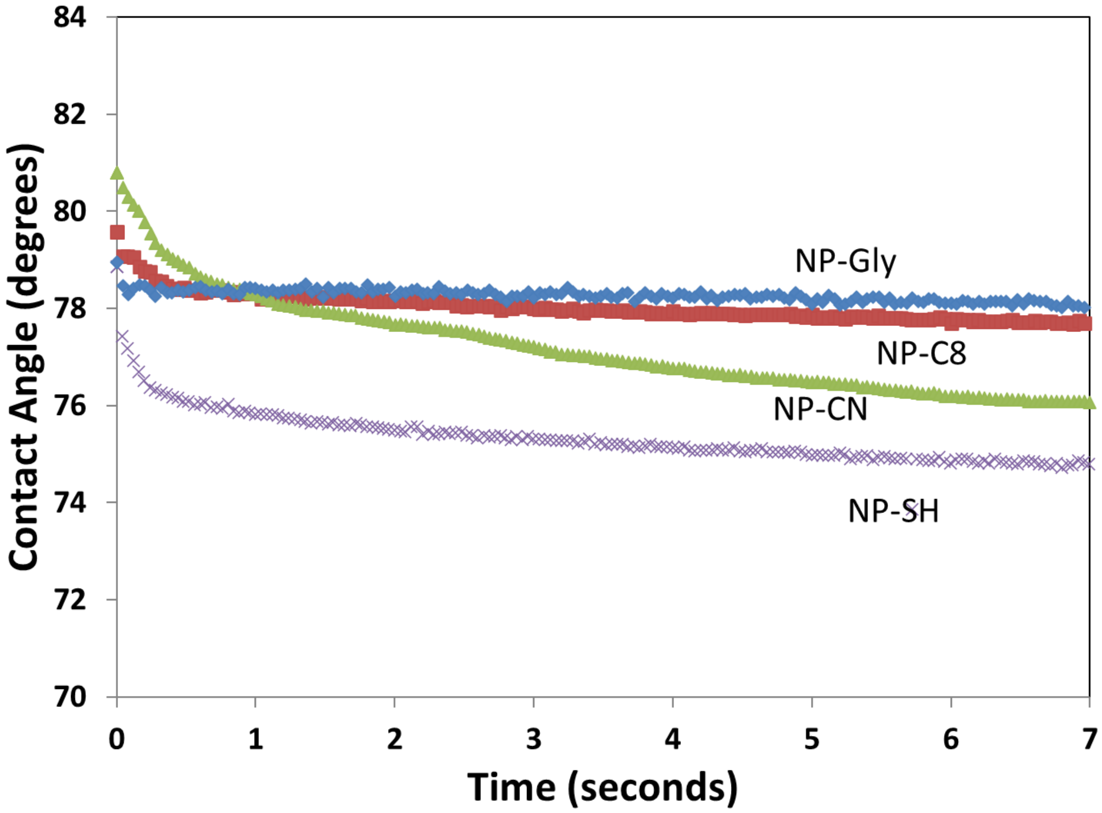

Figure S1. Evolution of the contact angle of a water sessile drop on the smooth side of the 2D film.

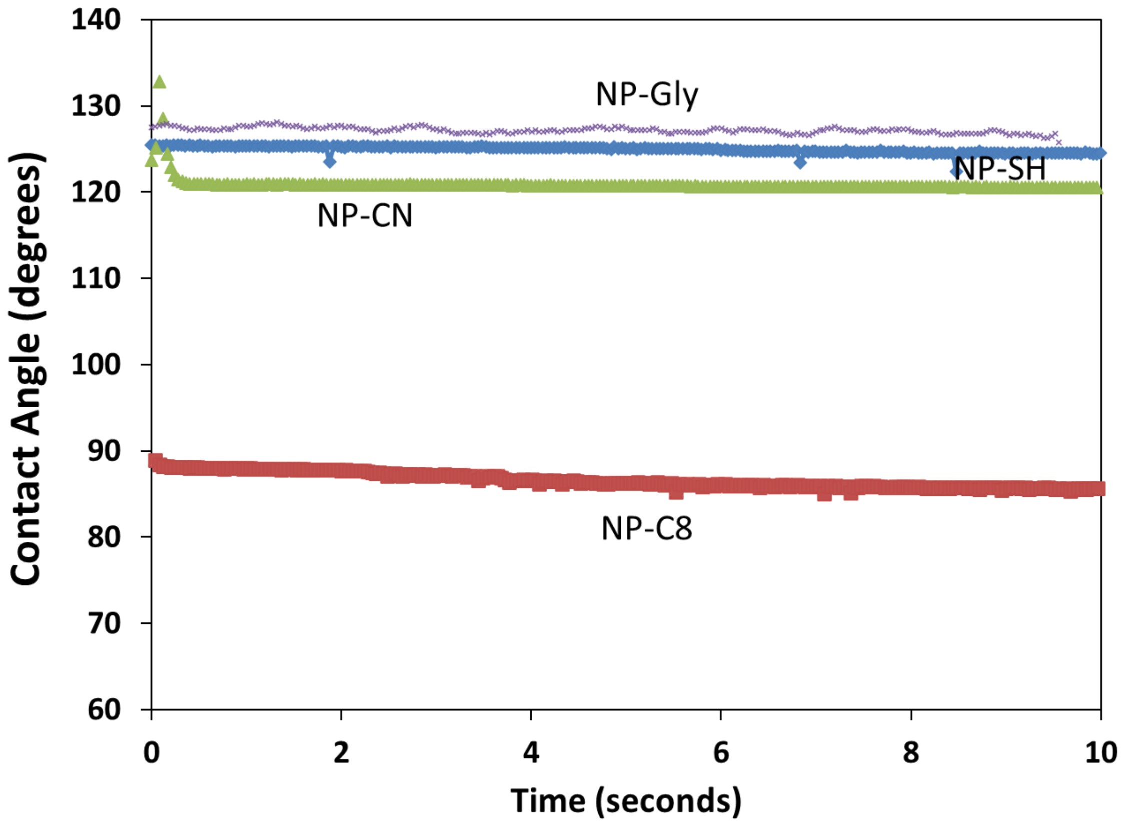

Figure S2. Evolution of the contact angle of a sessile water droplet on the nanostructured side of the HMDS-treated 2D film.
